# Unraveling Fish Community Diversity and Structure in the Yellow Sea: Evidence from Environmental DNA Metabarcoding and Bottom Trawling

**DOI:** 10.3390/ani15091283

**Published:** 2025-04-30

**Authors:** Jinyong Zhang, Xiaoyu Cui, Lin Lin, Yuan Liu, Jinqing Ye, Weiyue Zhang, Hongjun Li

**Affiliations:** 1 State Environmental Protection Key Laboratory of Coastal Ecosystem, National Marine Environmental Monitoring Center, Dalian 116023, China; yaliu@nmemc.org.cn (Y.L.); jqye@nmemc.org.cn (J.Y.); 2 College of Aquatic and Life Sciences, Dalian Ocean University, Dalian 116023, China; 15724585326@163.com; 3 Key Laboratory of South China Sea Fishery Resources Exploitation and Utilization, Ministry of Agriculture and Rural Affairs, South China Sea Fisheries Research Institute, Chinese Academy of Fishery Sciences, Guangzhou 510300, China; lonelydancer@sina.com; 4 College of Marine Ecology and Environment, Shanghai Ocean University, Shanghai 201306, China; zwy2017506@163.com

**Keywords:** eDNA metabarcoding, fish community, diversity, bottom trawling, Yellow Sea

## Abstract

The use of environmental DNA (eDNA) metabarcoding to analyze fish species diversity across different aquatic ecosystems is well documented. Nonetheless, there is a gap in validating eDNA metabarcoding studies on the diversity and structure of fish communities in coastal ecosystems, particularly in comparing these findings with bottom trawl catch data. In this study, we employed eDNA metabarcoding to explore species composition and relative abundance in fish communities, taxonomic-level diversity variations, and the interplay between community structures and environmental factors in the Yellow Sea and compared these results with those obtained from bottom trawl catches. In addition, we compared the various methods used to estimate the distributions of taxonomic, phylogenetic, and functional diversity factors. We found that eDNA metabarcoding detected a greater number of species (86 vs. 41), genera (73 vs. 37), and families (42 vs. 25) than bottom trawl results at each sampling station. eDNA metabarcoding provided higher Shannon, Simpson, and Chao1 alpha diversity indices than the bottom trawl results. The PCoA results showed that eDNA metabarcoding samples could be more clearly separated at the sampling sites in the Zhuanghe (ZH) and Lianyungang (LYG) areas than bottom trawling samples. The RDA analysis indicated that temperature, along with NO3- and NH_4_^+^ concentrations, were pivotal in shaping the geographical patterns of fish communities, as identified through eDNA metabarcoding, echoing findings from bottom trawling studies. Furthermore, our findings suggest that eDNA barcoding surpasses bottom trawling in detecting taxonomic and phylogenetic diversity, as well as in uncovering greater functional diversity at the local level. Conclusively, eDNA metabarcoding emerges as a valuable complement to bottom trawling, offering a multifaceted approach to biodiversity monitoring that not only boosts efficiency but also reduces environmental impact on coastal ecosystems.

## Simple Summary

We used environmental DNA (eDNA) metabarcoding and bottom trawl surveys to assess fish community diversity and structure in the Yellow Sea. eDNA metabarcoding detected significantly higher species richness (86 vs. 41 species), alpha diversity (Shannon, Simpson, and Chao1 indices), and taxonomic/phylogenetic/functional richness than bottom trawling. The PCoA results revealed clearer geographic clustering in eDNA data, while the RDA analysis identified temperature, NO_3_^−^, and NH_4_^+^ as key environmental drivers for both methods. eDNA captured more biodiversity components and local functional richness, demonstrating its potential to complement trawling for efficient, non-invasive coastal ecosystem monitoring.

## 1. Introduction

Monitoring and assessing changes in biodiversity is the basic premise in the protection of coastal ecosystems [1]. Today, the diversity of fish species serves as a pivotal indicator for assessing the health of aquatic ecosystems [2]. Additionally, the diversity of local fish species is essential for ecological insights, as well as for the management and preservation of coastal ecosystems. Traditional fish species diversity monitoring is mainly performed by hydro-acoustic surveys, electro-fishing, net fishing, and the use of traps to collect and identify fish species using morphological features and to count and weigh them to assess fish abundance and biomass [3,4]. However, these methods have many disadvantages, including habitat destruction, high time and effort costs, difficulty in morphological identification, and low capture rates of rare species [5]. Environmental DNA (eDNA) metabarcoding has the advantages of non-destructive sampling, simple and efficient operation, and high detection sensitivity [6]. It removes many of the shortcomings (monitoring biodiversity, managing invasive species, and supporting fisheries management) of traditional morphological monitoring methods and has great potential for biodiversity assessment applications [7,8,9,10,11].

Previous research has demonstrated the efficacy of eDNA metabarcoding as a robust method for surveying fish populations across a variety of ecosystems such as reservoirs [12,13], lakes [14,15], rivers [16,17,18], estuaries [2,19,20,21], coral reefs [22,23], mangrove forests [24], and oceans [25,26]. eDNA metabarcoding method, through non-invasive sampling, analyzes environmental DNA shed by organisms, reflecting its application in biodiversity research. [27,28]. The fish eDNA metabarcoding method demonstrates resilience to biological influences, including avoidance behavior, habitat specificity, and uneven distribution, unlike traditional methodologies. However, the eDNA metabarcoding method faces challenges, including inference biases and the limited availability of barcode and reference databases [29,30]. Consequently, integrating eDNA metabarcoding with traditional methods has been recommended for a more thorough and precise understanding of fish community compositions [31,32].

To date, previous research comparing eDNA metabarcoding with trawling techniques has primarily concentrated on examining taxonomic biodiversity. This includes analyses of the variety and specific preferences of taxa identified by each method [33,34,35]. Gaining a full perspective on biodiversity requires more than just tallying species numbers and identifying them taxonomically; it necessitates incorporating insights into their evolutionary backgrounds and the inherent biological and ecological attributes that define them, including aspects of phylogeny and functional traits [36,37]. The assessment of functional diversity relies on analyzing species traits, encompassing morphological, physiological, behavioral, and phenological characteristics observable at the individual level. These traits influence fitness through their impact on growth, reproduction, and survival, thereby shaping ecosystem functionality [38]. Phylogenetic diversity measures a community’s evolutionary history, represented by a phylogenetic tree that encapsulates morphological, anatomical, or genetic variations between taxa. Yet, it remains uncertain if eDNA metabarcoding and trawl capture techniques yield congruent or divergent insights into the functional and phylogenetic dimensions of biodiversity.

The Yellow Sea is a semi-enclosed shallow sea located on the edge of China’s continental shelf. It is an important northern Chinese fishing area and one of the world’s 50 Large Marine Ecosystems. The temperature and salinity of the Yellow Sea exhibit significant regional differences, with distinct characteristics of a marginal sea. From south to north and from the central part of the sea to the coast, both temperature and salinity decrease almost uniformly. In recent years, increasingly frequent human activity and serious environmental pollution have greatly affected the ecological balance of the Yellow Sea, and its biodiversity has seriously declined. Shan et al. (2014) conducted statistics on the results of fishery resources surveys in four seasons in the southern Yellow Sea and found that there were significant seasonal changes in the fishery resources structure from north to south in the southern Yellow Sea, which were closely related to bottom temperature and salinity [39]. In the coastal waters of the northern Yellow Sea, influenced by the alternating effects of the Yellow Sea coastal current, the Liaoning southern coastal current, and the Yellow Sea warm current, the seasonal changes in sea surface temperature are significant and salinity is relatively low. The dominant species of the fishery biological community are mainly demersal and warm–temperate fish, with a certain degree of seasonal succession [40].

We focused on the Yellow Sea to evaluate the use of eDNA metabarcoding compared to bottom trawling to study fish community composition and diversity in marine ecosystems. We examined the differences between fish communities in the northern and southern waters of the Yellow Sea, and compared the results obtained by eDNA metabarcoding methods and bottom trawling to verify the applicability of eDNA analysis to coastal fish community monitoring. We also compared the two methods with regard to their assessments of the distribution of three complementary biodiversity components—taxonomic, phylogenetic, and functional diversity—with a view to applying eDNA metabarcoding to multicomponent biodiversity assessments to develop a more holistic perspective on biodiversity.

## 2. Materials and Methods

### 2.1. Study Area and Sample Collection

**Figure 1:**
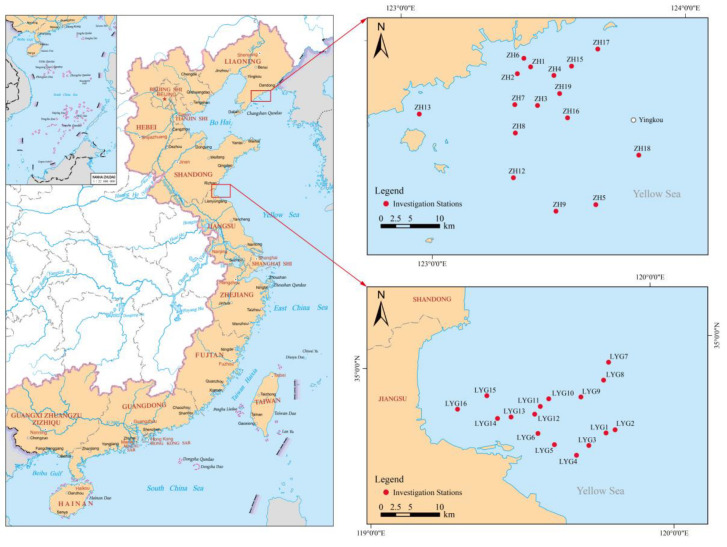
Distribution of sampling stations in the Yellow Sea. A total of 32 stations were investigated: 16 in the Zhuanghe (ZH) coastal area and 16 in the Lianyungang (LYG) coastal area.

In April 2023, we established two sets of 16 sampling stations in the Zhuanghe area (ZH) in the North Yellow Sea and the Lianyungang area (LYG) in the South Yellow Sea (Figure 1, Supplementary Table S1). Five-liter surface water samples were collected in sterile polyethylene buckets at 32 bottom trawling stations for eDNA metabarcoding analysis. After collection, the water samples were stored on ice and brought to the laboratory within 12 h for further processing. Bottom trawl sampling began after the water samples and environmental data had been collected at each station. After preliminary classification, the fish collected in the bottom trawl catches were frozen and stored in refrigerators in the ship’s cabin, brought to the laboratory, and stored at −20 °C. The fish species were identified using morphological characteristics, based on *Fishes of the Bohai Sea and Yellow Sea* [41] and the *Checklist of Marine Biota of China Seas* [41,42].

A further 500 mL water sample was collected from each station, stored in a sterilized sample bottle, and brought back to the laboratory for analysis. Environmental factors related to ammonia nitrogen (NH_4_^+^), nitrite (NO_2_^−^), nitrate (NO_3_^−^), and phosphate (PO_4_^3−^) were determined within 24 h according to national standard methods.

### 2.2. DNA Extraction, PCR Amplification, and High-Throughput Sequencing Analysis

All of the equipment was routinely disinfected with UV light and bleach in a sterile laboratory, and DNA was extracted using a PowerWater^®^DNA Isolation Kit (MOBIO, Jefferson City, MO, USA) according to the manufacturer’s instructions. After extraction, a NanoDrop One spectrophotometer (Thermo Fisher Scientific, Carlsbad, CA, USA) was used to determine the concentration and quality of the DNA isolates. The eDNA template was amplified by PCR, using the fish mitochondrial 12S rRNA gene primers (MiFish-E-F: 5′-GTTGGTAAATCTCGTGCCAGC-3′ and MiFish-E-R: 5′-CATAGTGGGGTATC TAATCCTAGTTTG-3′) [43] with a 8 bp barcode, and the amplification length was found to be ~170 bp. PCR reactions were performed in triplicate 20 μL mixture containing 4 μL of 5 × FastPfu Buffer (TransGen Biotech, Beijing, China), 2 μL of 2.5 mM dNTPs, 0.8 μL of each primer (5 μM), 0.4 μL of FastPfu Polymerase (TransGen Biotech, Beijing, China), and 10 ng of template DNA. Amplification occurred as follows: initial denaturation at 95 °C for 5 min, denaturation at 95 °C for 30 s, annealing at 58 °C for 30 s, and extension at 72 °C for 45 s; 27 cycles were performed with a final extension at 72 °C for 10 min. The model of the PCR amplification instrument was ABI GeneAmp^®^ 9700 (Applied Biosystems, Foster, CA, USA). For each set of replications, ultrapure water was used as the substrate for negative control. The PCR products were extracted from 2% agarose gels and purified using the AxyPrep DNA Gel Extraction Kit (Axygen Biosciences, Union City, CA, USA) according to the manufacturer’s instructions. Purified PCR products were quantified by Qubit^®^3.0 (Life Invitrogen, Carlsbad, CA, USA). The pooled DNA product was used to construct Illumina pair-end library following Illumina’s genomic DNA library preparation procedure. All libraries were paired-end sequenced on an Illumina NovaSeq 6000 platform (Illumina, San Diego, CA, USA). The raw reads were deposited into the NCBl Sequence Read Archive (SRA) database (Accession Number: SRP46958372~SRP46958403).

### 2.3. Bioinformatics Analysis

The original sequences underwent filtration using Trimmomatic (v.0.36) [44] to remove low-quality sequences, and FLASH (v.1.2.11) [45] was utilized to combine paired reads into sequences. The samples were distinguished by the barcodes and primer regions at both ends of the sequences, and the sequence orientation was adjusted using QIIME 2 (v.2023.2) [46]. The reference sequences from the GOLD database was combined with Usearch software (v.11.0.667) [47], using the “uchime3_denovo” and reference-based approach to remove chimeras. The Usearch (v.11.0.667) was used to cluster the effective tags into operational taxonomic units (OTUs) with a similarity threshold of ≥97% [35,48,49,50]. 

The Brocc annotation algorithm was used to conduct species taxonomy annotation for the obtained OTUs sequences [51]. All of the fish listed in the Yellow and Bohai Sea historical records were used to build a local database. The annotation results were manually reviewed to eliminate non-fish information, and OTUs with identity > 97%, E-value < 10^−5^, and coverage > 90% were selected for comparison with fish. The OTUs that had the same species identification result were merged. The obtained OTUs were sequentially compared with GenBank database [52] and MitoFish database [53].

### 2.4. Statistical Analysis

In this study, we utilized the alpha diversity index to discern variations in fish community compositions identified by the two methods; four diversity indices were selected for species diversity calculation, namely, the Shannon index, Simpson index, Pielou’s Evenness index (Pielou_J), and Chao1. Beta diversity is a core indicator used in ecology to measure the differences in species composition between different communities, reflecting the spatial or temporal changes in biodiversity. Subsequent analyses standardized raw data from bottom trawling and eDNA metabarcoding via the Hellinger transformation [17], employing R (v.4.2.0) for analysis [54]. We computed pairwise compositional distances among stations utilizing the Bray–Curtis metric, using the vegan R package (function: “vegdist”) [55]. Spatial patterns in fish communities were examined through Principal Coordinate Analysis (PCoA), applying the Bray–Curtis dissimilarity metric via the R package statistics (function: “cmdscale”). The significance of the result was assessed with Analysis of Similarities (ANOSIM), employing the vegan R package (function: “anosim”) [55].

Datasets representing environmental factors underwent natural-log transformation via log1p, while abundance datasets were standardized using the Hellinger transformation. Post-normalization, a redundancy analysis (RDA) was performed to explore and depict the relationships between fish communities and their environment, utilizing the vegan R package (function: “rda”) [55]. Subsequently, the R2 value was adjusted through the vegan R package (function: “RsquareAdj”) [55]. Significance testing for each environmental factor was conducted using the vegan R package (function: “envfit”), following 999 random permutations. Differences were considered significant when *p* < 0.05.

The taxonomic richness of each sampling site was assessed through the analysis of a refined taxa table, which included 110 taxa (refer to Supplementary Table S2). For assembling functional traits pertinent to crucial ecosystem functions, data on eight characteristics were sourced from the FishBase database [56]: maximum length, trophic level, water column position, depth range, body shape, reproductive mode, fertilization mode, and type of parental care (see Supplementary Table S3). Phylogenetic diversity (PD) indices were determined using 100 species-level phylogenetic trees to incorporate phylogenetic uncertainty. The standardized effect size (SES) quantifies the difference between an observed phylogenetic/functional index of diversity and an expected distribution of the same diversity index under a null model of random association of taxa with their phylogenetic relationships or biological traits. Assuming normality, SES values > 1.96 indicate significant over-dispersion at a 5% test level, while SES values < −1.96 indicate significant spatial clustering of species with certain traits. This methodology echoes the phylogenetic/functional index of diversity approaches adopted in earlier research [57,58,59].

## 3. Results

### 3.1. Composition and Relative Abundance of Fish Communities in the Yellow Sea

**Figure 2:**
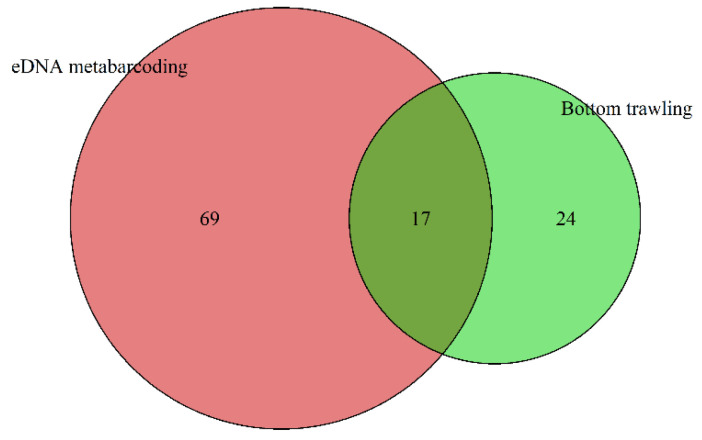
Number of fish species identified by eDNA metabarcoding and bottom trawling: 69 taxa were only detected by eDNA and 23 taxa were only detected by bottom trawling, while 17 taxa were detected by both methods.

A total of 3,941,101 reads were obtained from 32 sampling stations in the ZH and LYG coastal areas using 12S rRNA high-throughput sequencing. A total of 1374 OTUs were detected by annotating and classifying the OTUs sequenced, and 86 species of fish belonging to 25 orders, 42 families, and 73 genera were identified. A total of 5110 fish were collected by bottom trawling in the ZH and LYG coastal areas, and 41 species were identified, belonging to 11 orders, 25 families, and 37 genera. When the eDNA metabarcoding and bottom trawling datasets were combined, 110 fish species from 26 orders, 51 families, and 89 genera were identified (Supplementary Table S2). Compared with the bottom trawling results, greater numbers of species, genera, families, and orders were detected by eDNA metabarcoding. Seventeen fish (15.45%) species were identified by both eDNA metabarcoding and bottom trawling, and an additional 69 (62.73%) and 24 (21.82%) species were detected by eDNA metabarcoding or bottom trawling alone, respectively (Figure 2).

**Figure 3:**
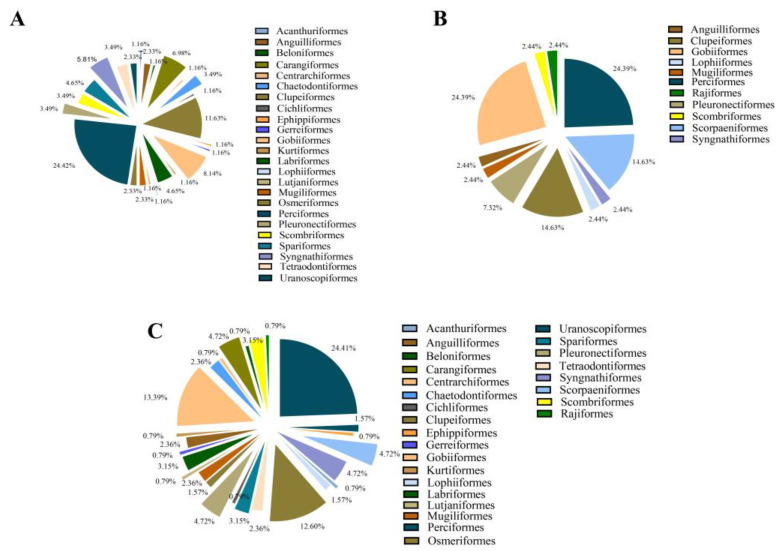
Order of occurrence by species frequencies for (**A**) eDNA metabarcoding; (**B**) bottom trawling; and (**C**) both methods. The mean frequencies of fish observed (computed from number of individuals) across the study area are given in percentages.

Among the 1374 OTUs obtained by eDNA metabarcoding, Perciformes had the largest number of species, with a total of 21 species, accounting for about 24.42% of the total fish species detected. Next were Clupeiformes and Gobiiformes, accounting for 11.63% and 8.14%, respectively (Figure 3A). Of the 5110 fish captured by the bottom trawling method, Perciformes (24.39%), Gobiiformes (24.39%), Clupeiformes (14.63%) and Scorpaeniformes (14.63%) were the four dominant orders identified (Figure 3B). Combining the bottom trawling and eDNA metabarcoding datasets revealed that the top three orders were Perciformes (24.41%), Gobiiformes (13.39%), and Clupeiformes (12.60%) (Figure 3C). Nine orders were found by both methods: Anguilliformes, Clupeiformes, Gobiiformes, Lophiiformes, Mugiliformes, Perciformes, Pleuronectiformes, Scombriformes, and Syngnathiformes (Figure 3A–C).

Based on the relative abundance of eDNA metabarcoding records at each sampling station, the top 10 dominant fish species by sequence abundance accounted for 81% of the total number of reads assigned to species (Supplementary Figure S1A). Of the 5110 fish caught by bottom trawling, the top 10 species by sequence abundance accounted for 92% of the total catch (Supplementary Figure S1B).

### 3.2. Alpha and Beta Diversity of Fish Communities in the Yellow Sea

The mean Shannon diversity index using eDNA metabarcoding was 2.16 (0.31–3.38), and the value for bottom trawling was 1.50 (0.52–2.41). The mean Simpson diversity index for eDNA metabarcoding was 0.77 (0.09–0.96), and that for bottom trawling was 0.67 (0.19–0.88). The mean Pielou_J index for eDNA metabarcoding was 0.59 (0.09–0.92), and the mean for bottom trawling was 0.69 (0.24–0.96). The average Chao1 index for eDNA metabarcoding was 41 and that for bottom trawling was 12 (Supplementary Table S4).

**Figure 4:**
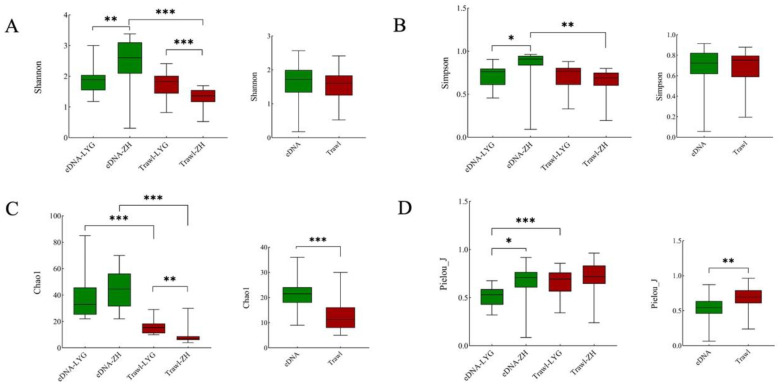
Comparison of fish alpha diversity indices identified by eDNA metabarcoding and bottom trawling. The Shannon diversity index (**A**), Simpson diversity index (**B**), Chao1 index (**C**) and Pielou_J index (**D**) between the results obtained from eDNA metabarcoding and bottom trawling methods in both the ZH and LYG areas. Note: ***: *p* < 0.001, **: *p* < 0.01, *: *p* < 0.05.

The Shannon, Simpson, and Chao1 indices for eDNA metabarcoding were all higher than those for the bottom trawling method, indicating that eDNA metabarcoding can obtain higher fish species diversity. Further analysis showed significant differences in the Shannon and Simpson diversity indices between the eDNA metabarcoding and bottom trawling methods in ZH (*p* < 0.05, Figure 4A,B). There were significant differences in the Pielou_J index between the eDNA metabarcoding and bottom trawling methods in LYG (*p* < 0.05, Figure 4D), and significant differences in the Chao1 index for both ZH and LYG (*p* < 0.05, Figure 4C).

**Figure 5:**
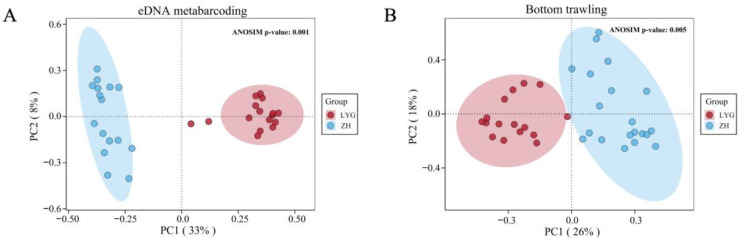
PCoA diagram of fish beta diversity of all stations based on eDNA metabarcoding (**A**) and bottom trawling (**B**).

Based on the PCoA analysis of the sequence abundance of fish species shown in Figure 5A, when eDNA metabarcoding was used to detect the spatial distribution of fish in the Yellow Sea, PC1 explained 33% of the variation in the PCoA results based on Bray–Curtis distance, while PC2 explained 8%. The ZH and LYG coastal areas formed separate sets in the PCoA space, and the 16 stations in each area were relatively close to each other, showing a trend towards independent aggregation. In the PCoA analysis results using bottom trawling (Figure 5B), PC1 explained 26% of the variation, and PC2 explained 18%. Although the stations in ZH and LYG also showed a trend towards independent aggregation, the two areas formed an intersecting set in the PCoA space, and the distance between the stations was greater. The results showed that eDNA metabarcoding samples could be more clearly separated in the ZH and LYG areas than those obtained by bottom trawling.

### 3.3. Relationship Between Fish Community Composition and Environmental Factors

Supplementary Table S5 shows that the environmental factors, Temp (temperature), and Nitrate (NO_3_^−^) and Ammonium (NH_4_^+^) levels had extremely significant influences on the species ranking results (*p* < 0.01).

**Figure 6:**
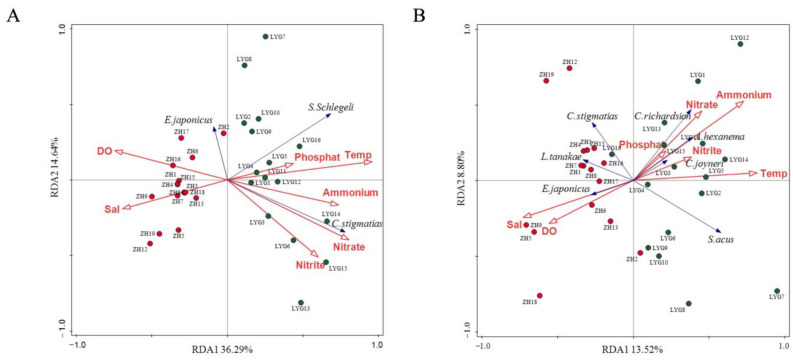
Relationships between fish community composition, based on eDNA metabarcoding (**A**) and bottom trawling (**B**) and the environmental factors. The red arrows represent environmental factors, while the blue arrows indicate representative fish species. Red circle markers are used for the stations in the ZH area, while dark green circle markers are used for the stations in the LYG area.

In the RDA analysis of eDNA metabarcoding, the amount of variation explained by the RDA1 and RDA2 axes was 36.29% and 14.64%, respectively (Figure 6A). The two subsamples of ZH and LYG showed high discretization, although the discretization between the samples from ZH was lower than that from LYG. The ray angles between most ZH samples and salinity (Sal) and dissolved oxygen (DO) were acute, indicating that most samples in ZH were positively correlated with Sal and DO. Most samples from the LYG area were positively correlated with Temp. The RDA diagram also shows that Temp was positively correlated with NH_4_^+^, NO_2_^−^, NO_3_^−^, and PO_4_^3−^, while Temp and DO were negatively correlated with Sal. The RDA analysis of the bottom trawl samples showed that the RDA1 and RDA2 axes explained 13.52% and 8.80% of the variation, respectively (Figure 6B). The RDA analysis results showed that the sampling stations in ZH and LYG also showed high discretization, and that Temp had a significant negative influence on Sal and DO, while it was positively correlated with NH_4_^+^, NO_2_^−^, NO_3_^−^, and PO_4_^3−^, consistent with the RDA analysis results for eDNA metabarcoding.

### 3.4. Spread and Variation of Taxonomic, Phylogenetic, and Functional Diversity

**Figure 7:**
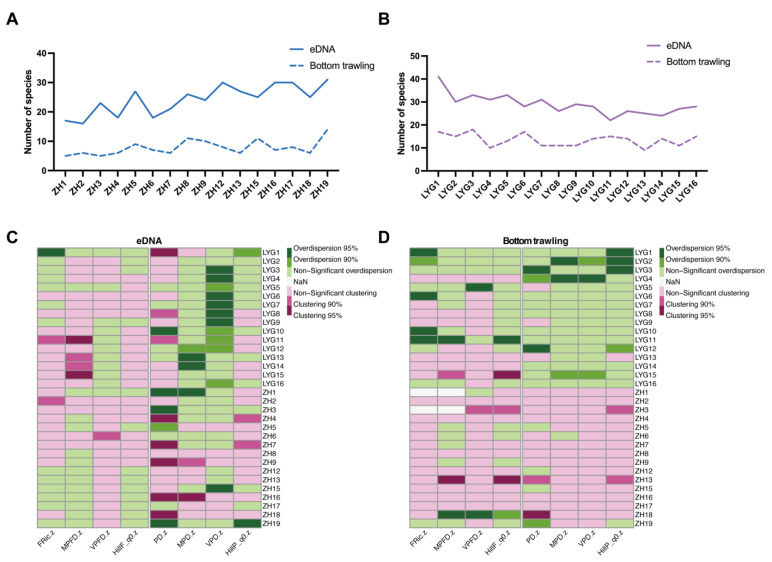
Visualization of the biodiversity indicators. The taxonomic richness measured by eDNA and bottom trawling in both the ZH (**A**) and LYG (**B**) stations; The phylogenetic diversity, and functional diversity, measured by eDNA (**C**) and bottom trawling (**D**) at each sampling station.

Across all sampling sites, eDNA consistently identified greater taxonomic richness compared to bottom trawl collections (Figure 7, Supplementary Table S6). eDNA samples captured an average of 26.60 taxa, while bottom trawl samples captured 10.60. The SES for the PD index were notably higher in eDNA samples, indicating trends of over-dispersion (SES.PD = 0.96 ± 0.8) and clustering (SES.PD = −0.33 ± 1.09) for eDNA metabarcoding and bottom trawling sampled communities, respectively. In eDNA metabarcoding, five out of 32 stations exhibited PD values significantly above the null model’s predictions (LYG10, ZH1, ZH3, ZH7, and ZH19), whereas six stations demonstrated significantly lower PD values (LYG1, LYG8, LYG11, ZH9, ZH11, and ZH16). Regarding bottom trawling, three stations (LYG3, LYG4, and LYG12) recorded PD values exceeding those of the null model, with no stations showing lower values. Considering functional diversity, none of the FD values of stations sampled by eDNA metabarcoding deviated from the null model. For bottom trawling, two stations (LYG11 and ZH18) had a higher FD than the null model, and three stations (LYG15, ZH3, and ZH13) had a lower FD than the null model.

Further analysis showed that (Figure 7C), in terms of functional richness, LYG1 had the highest SES. The eDNA samples exhibited pronounced functional diversity and volume, significantly surpassing expectations based on the null model, thus suggesting notable functional over-dispersion. On the contrary, stations LYG11 and ZH2 demonstrated the least functional richness according to SES.FRic measurements, signifying considerable functional clustering. At these locations, the species, being closely related, were uniformly distributed across the functional spectrum, as indicated by low SES.VPFD scores. Specifically, stations LYG11, LYG13, LYG14, and LYG15 exhibited notably clustered SES.MPFD values, recorded at 0.50, 0.09, 0.16, and 0.51, respectively. In terms of regularity, these stations exhibited some over-dispersion, although not significantly so (SES.VPFD between 0.49 and 0.56). However, station ZH6, in the northern Yellow Sea, was significantly clustered, with SES.VPFD reaching 2.13. The other northern Yellow Sea stations were also clustered, although none of them significantly so. Regarding the components of phylogenetic diversity, the SES.MPD and SES.VPD indicators yielded comparable outcomes across all sampling sites, with a consistency rate of 71.9% and 81.3% of the stations showing over-dispersion in these metrics, respectively. The average pairwise distances among species on the phylogenetic tree were notably high and varied, indicating the coexistence of species from highly distinct phylogenetic branches alongside several species that are phylogenetically similar (within the same lineage). Stations exhibiting the highest SES.MPD and SES.VPD values, indicating significant phylogenetic over-dispersion, were predominantly found in the LYG region, including LYG8, LYG9, LYG13, and LYG14. Conversely, the lowest values for SES.MPD and SES.VPD were observed in the northern part of the ZH region, such as ZH16, and were significantly clustered.

## 4. Discussion

### 4.1. eDNA Metabarcoding as a Promising Tool for Assessing Fish Biodiversity in the Yellow Sea

In the present study, the species detection rate of eDNA metabarcoding was 78.18% (86 species), compared with 37.27% (41 species), for bottom trawl sampling, indicating that eDNA metabarcoding was superior to bottom trawling in detecting fish species richness. Acanthuriformes, Beloniformes, Centrarchiformes, Cichliformes, Ephippiformes, Gerreiformes, Kurtiformes, Lophiiformes, and Lutjaniformes species were all detected.

According to the Shannon and Simpson indices, eDNA metabarcoding detected higher alpha diversity than the bottom trawling method, indicating that eDNA metabarcoding could more comprehensively reflect fish species richness and evenness. The traditional bottom trawling method exhibited poor applicability in rocky reef areas, resulting in the failure to capture several fish species such as *Sillago sihama*, *Rhabdosargus sarba*, *Seriola dumerili*, and *Siganus fuscescens* via bottom trawling, while they were only detected by eDNA metabarcoding. The vast area of the Yellow Sea gives rise to significant variations in habitat heterogeneity, seabed topography, and distribution of seasonal currents and water masses. Traditional trawl samples were difficult to perform, resulting in failure to catch all of the fish species present, thus underestimating their community diversity [60]. On the other hand, eDNA sampling is spatially fluid and can integrate biodiversity over small spatial and temporal scales and so circumvent the effects caused by differences in habitat type and fish behavior [61,62]. eDNA metabarcoding demonstrated superiority over bottom trawling in sampling pelagic and mid-water fish species. Species including the *Coilia mystus*, *Planiliza haematocheilus*, *Scomberomorus niphonius*, *Nuchequula nuchalis*, *Sardinella hualiensis*, and *Tylosurus crocodilus* were solely detected by eDNA metabarcoding. However, it is worth noting that eDNA metabarcoding methods may also overestimate some low-abundance groups. Previous experiments showed that eDNA persisted for 72 h–21 days post-species removal from water [63,64,65], and fish DNA fragments can spread to adjacent sampling stations through water currents [66]. In contrast, bottom trawling data only provides information on the fish actually present in the immediate sampling area.

Evaluating fish species diversity necessitates data on species presence or absence as well as their respective abundances [16,67,68]. Earlier research has established a positive correlation between abundance/biomass and eDNA concentration, although these studies were primarily performed under controlled indoor conditions [69] or in freshwater environments [70,71]. However, the main challenge hindering eDNA applicability has been the inability to infer absolute species abundances from multispecies analysis [4]. Shelton et al. (2023) developed a model to estimate initial DNA proportions in metabarcoding samples while accounting for species-specific amplification biases by sequencing mock community samples of known concentration of DNA extracts for a given list of taxa alongside environmental samples [72]. Ledger et al. (2024) provided a research framework for estimating the absolute eDNA concentrations and biomass of multiple species by designing species-specific metabarcoding primers and quantifying metabarcoding data, including correcting for PCR biases [73]. Guri et al. (2024) developed a Bayesian joint model for analyzing eDNA data in conjunction with other ecological data sources, such as trawl catch data, to improve the quantitative accuracy of metabarcoding analyses [4]. These studies bridged the gap between eDNA and trawl surveys, quantifying their bias (trawl catchability, DNA shedding, degradation, dilution, transport, recovery rate, and isolation efficiency) alongside the biological relationships between fish abundance and DNA concentration in the marine environment.

### 4.2. Characteristics of Fish Community Structure in the Yellow Sea

According to survey data from the Yellow Sea during the 1950s and 1960s, the fishery species were mainly high-quality bottom- and near-bottom-dwelling fish with high economic value, such as *Larimichthys polyactis*, *Trichiurus lepturus*, and flatfishes [74]. In the 1970s and 1980s, *Clupea pallasii*, *Scomberomorus niphonius*, and *Scomber japonicus* successively became the dominant species [75]. By the end of the 1990s, the low-value fish Lophius litulon and Liparis tanakae gradually became the dominant species [76]. In this study, based on a combination of eDNA metabarcoding and bottom trawling, we found that the dominant species in the Yellow Sea were *Chaeturichthys stigmatias*, *Engraulis japonicus*, *Callionyuus richardsion*, *Syngnathus acus*, *Cynoglossus joyneri*, *Liparis tanakae*, and *Amblychaeturichthys hexanema*. Compared with the historical data, the dominant fishery species in the Yellow Sea have changed greatly, and high-quality bottom- and near bottom-dwelling fishes with high economic value, such as *Larimichthys polyactis*, *Hexagrammos otakii*, *Pseudopleuronectes yokohamae*, *Kareius bicoloratus*, and *Sebastes schlegeli* have been replaced. The economically important Yellow Sea fishery species *Larimichthys polyactis* occurred in only two of the 32 stations in this survey. Its dominance was low, and its population showed signs of continual decline.

Fish community structures differ in different regions, and differences in their composition play a crucial role in maintaining ecosystem functions [77,78]. In this study, PCoA analysis of eDNA metabarcoding results showed that ZH and LYG formed disjoint sets in the PCoA space, and that the 16 stations in each area were relatively close to each other, showing independent aggregation trends. The results showed that the fish community structures of ZH and LYG were very similar between stations in the two areas, and that the spatial distribution of fish community structures was significantly different between the North and South Yellow Seas. This may be due to the geographical distance between the North Yellow Sea and the South Yellow Sea, and the large differences in habitats (rivers, coastal currents, sediment types, etc.), resulting in different biogeographic distribution patterns of fish communities [74].

### 4.3. Effects of Environmental Factors on Fish Community Structure in the Yellow Sea

Our study employed redundancy analysis (RDA) to assess how environmental factors influence fish community composition variations. The structure of the fish community was influenced by a variety of environmental aspects, including water quality, habitat condition, surrounding land use, and hydrological features [79,80,81]. For example, water temperature influences fish metabolic rates, whereas salinity impacts their respiratory metabolism and digestion efficiency [82]. DO can directly affect the behavior of fish, such as feeding, growth, reproduction, and distribution [82,83]. The growth, development, survival, and diversity of marine organisms are strongly influenced by changes in marine environmental factors [84]. In a study of the coastal waters of the northern Yellow Sea, Cui et al. (2023) found that sea surface temperature, sea bottom temperature, sea bottom salt, and chlorophyll levels are the main environmental factors leading to temporal and spatial changes in fish community structure, among which the effects of sea surface temperature and sea bottom salt are particularly significant [40].

The RDA results based on eDNA metabarcoding and bottom trawling data showed that Temp, NO_3_^−^, and NH_4_^+^ levels emerged as the primary environmental influencers on fish communities in the Yellow Sea, with the RDA analysis accounting for 50.93% of the variance in community structure (Figure 6A). The occurrence of *E. japonicus* was positively correlated with Sal and DO, and negatively correlated with NO_2_^−^, NO_3_^−^, and NH_4_^+^. It is a warm-water pelagic fish with a high dissolved oxygen demand. Zhu et al. (2021) found that increasing DO content was associated with an increasing trend in *E. japonicus* density, mainly concentrated in sea areas with DO higher than 8 mg/L [85]. NH_4_^+^ and total nitrogen are indicators reflecting different forms of nitrogen in the water, and the total phosphorus level reflects the total amount of various phosphorus compounds in the water. Excessive total phosphorus and total nitrogen levels are important causes of water eutrophication. Therefore, total phosphorus and total nitrogen levels affect fish distribution to a certain extent. The distributions of *C. stigmatias* and *Syngnathus schlegeli* were positively correlated with NO_2_^−^, NO_3_^−^, NH_4_^+^, Temp, and PO_4_^3−^, and were negatively correlated with Sal and DO. Studies have shown that *C. stigmatias* is a bottom-dwelling warm-water species in coastal and estuarine waters, possibly due to the food-rich environment resulting from the interaction between nutrients (nitrogen, phosphorus, etc.) carried into the sea by runoff and marine environmental variables [86,87]. The eDNA metabarcoding method combined information on fish distribution and environmental factors and provided a scientific basis for the monitoring and evaluation of fish biodiversity in the Yellow Sea.

### 4.4. Fine-Scale Diversity Patterns

In this study, we focused not only on taxonomic diversity but also on documented data on the functional and phylogenetic diversity shown by both methods. Locally, eDNA metabarcoding surpassed bottom trawling in uncovering taxonomic diversity, requiring an average of 2.51 trawling attempts to match the taxonomic richness captured by eDNA metabarcoding. Consistently, earlier research has found that eDNA metabarcoding identifies a broader range of taxa compared to traditional approaches, and with reduced sampling effort [88], even in comparisons with trawling methods [25]. eDNA can detect certain species that are not generally detected by visual censuses (e.g., pelagic, mobile, or crypto-benthic species) [89,90]. In our research, eDNA metabarcoding captured a wide array of taxa, spanning a larger segment of the phylogenetic tree compared to trawling. The PD of species identified via eDNA was notably higher, and SES analyses revealed a trend of phylogenetic over-dispersion among these taxa, unlike the more phylogenetically concentrated species identified through trawling. Our findings align with Rozanski et al. (2022), who also observed phylogenetic over-dispersion in communities identified using eDNA [57,58]. At the station-specific level, bottom trawling revealed greater functional diversity at four locations (LYG10, LYG11, LYG12, and LYG16). However, when analyzing the data on a regional scale, eDNA metabarcoding surpassed bottom trawling in detecting functional richness, as the scope and diversity of the functional traits of taxa identified by eDNA were broader and more varied compared to those uncovered through bottom trawling.

Utilizing the eDNA metabarcoding technique, we advanced a comprehensive view of biodiversity by exploring three aspects of functional and phylogenetic diversity: richness, divergence, and regularity. Fish communities observed at the LYG stations exhibited enhanced species richness, pronounced phylogenetic over-dispersion, and reduced functional clustering across these dimensions compared to those recorded at the ZH stations. This may be due to the low number of species detected per station at the northern ZH stations, and the occurrence of numerous small to medium-sized demersal species (e.g., *Ammodytes personatus*, *Sardinella hualiensis*, *Pennahia pawak*, *Coilia mystus*). These species primarily stem from two dominant groups, Perciformes and Clupeiformes, leading to a reduced level of phylogenetic divergence and more variable phylogenetic distances in comparison to the LYG locations. Furthermore, functional clustering was more pronounced at the ZH sites, suggesting a higher degree of functional similarity among species, thereby increasing functional redundancy. While functional diversity was marginally higher at the LYG sites, the overall study area showed tendencies towards functional clustering. This implies that, despite the species at the LYG sites originating from widely varied phylogenetic lineages (indicative of phylogenetic over-dispersion), they exhibited common functional traits and fulfilled similar critical ecological roles to those observed at the ZH sites. This observation aligns with Rozanski et al. (2022) findings regarding fish biodiversity along the southern Brittany coast of the Iroise Sea, France [57].

## 5. Conclusions

This study juxtaposed eDNA metabarcoding against bottom trawling techniques to analyze the composition, relative abundance, diversity, and environmental affiliations of fish species communities in the Yellow Sea. eDNA metabarcoding surpassed bottom trawling in identifying a broader spectrum of families, genera, and species, thereby enhancing our understanding of biodiversity in the region and providing new and important data for the management and conservation of coastal marine ecosystems. This study also demonstrates that eDNA metabarcoding presents great potential for monitoring geographical variations in fish composition in coastal marine ecosystems. By integrating functional and phylogenetic information, eDNA metabarcoding technology can effectively recover multiple components of biodiversity at the regional scale, highlighting the north/south diversity gradient. The PCoA results showed that eDNA metabarcoding samples can be more clearly separated by ZH and LYG areas than bottom trawling. Despite the differences in results compared to traditional methods, eDNA metabarcoding can also reveal the impact of environmental factors on fish communities, which may be because environmental factors also influence the degradation rate of eDNA. In conclusion, this study demonstrates that eDNA metabarcoding, which is an effective approach that could augment bottom trawling, enhancing the ability to assess fish community structure in coastal ecosystems and enabling comprehensive biodiversity monitoring.

## Data Availability

The datasets presented in this study can be found in online repositories. The raw reads were deposited into the NCBl Sequence Read Archive (SRA) database, with access numbers SRP46958372 to SRP46958403.

## Supplementary Materials

The following supporting information can be downloaded at: https://www.mdpi.com/article/10.3390/ani15091283/s1, Figure S1: Relative abundance (%) of fish community members, at the species level, recorded by eDNA metabarcoding (A) or bottom trawling (B); Table S1: Water depth information at sampling stations in LYG and ZH area of Yellow Sea. Table S2: Composition of fish species in LYG and ZH of Yellow Sea via eDNA metabarcoding and bottom trawling; Table S3: Species traits used to calculate functional diversity; Table S4: Summary of alpha diversity indices; Table S5: Monte Carlo permutation test for RDA (conditional effect) based on eDNA metabarcoding data (A) and bottom trawling data (B); Table S6: Summary of the biodiversity indicators, taxonomic richness, phylogenetic diversity, and functional diversity, measured by eDNA and bottom trawling at each sampling station.
