# Supplementary material for: Unraveling Fish Community Diversity and Structure in the Yellow Sea: Evidence from Environmental DNA Metabarcoding and Bottom Trawling
Source: Animals (Basel). 2025 Apr 30;15(9):1283. doi: 10.3390/ani15091283 (PMC12070852; doi:10.3390/ani15091283)
Supplement: Supplementary file 1 [file animals-15-01283-s001.zip › Supplementary Table S1.pdf]

Supplementary Table S1: Water depth information at sampling stations in LYG and ZH area of Yellow Sea.

Station	Water depth (m)	Station	Water depth (m)
LYG1	17.6	ZH1	9.8
LYG2	17.3	ZH2	8.2
LYG3	12.7	ZH3	18.6
LYG4	12.7	ZH4	13.5
LYG5	8.1	ZH5	32.4
LYG6	8.2	ZH6	9.1
LYG7	21.7	ZH7	14.3
LYG8	21.2	ZH8	17.2
LYG9	17.4	ZH9	39.3
LYG10	14.7	ZH12	32.4
LYG11	12.5	ZH13	7.2
LYG12	10.7	ZH15	9.9
LYG13	11.1	ZH16	18.3
LYG14	10.2	ZH17	5.1
LYG15	13.1	ZH18	33.6
LYG16	7.1	ZH19	19.2
