# Supplementary material for: Unraveling Fish Community Diversity and Structure in the Yellow Sea: Evidence from Environmental DNA Metabarcoding and Bottom Trawling
Source: Animals (Basel). 2025 Apr 30;15(9):1283. doi: 10.3390/ani15091283 (PMC12070852; doi:10.3390/ani15091283)
Supplement: Supplementary file 1 [file animals-15-01283-s001.zip › Supplementary Table S2.pdf]

Supplementary Table S2: Composition of fish species in LYG and ZH of Yellow Sea via eDNA metabarcoding and bottom trawling.

No.	Order	Family	Genus	Species	eDNA metabarcoding		Bottom trawling		
					LYG	ZH	LYG	ZH	
1	Acanthuriformes	Acanthuridae	Ctenochaetus	*Ctenochaetus tominiensis*		+			
2	Anguilliformes	Muraenidae	Gymnothorax	*Gymnothorax javanicus*		+			
3	Anguilliformes	Ophichthidae	Pisodonophis	*Pisodonophis cancrivorus*		+			
4	Anguilliformes	Congridae	Conger	*Conger japonicus*			+		
5	Beloniformes	Belonidae	Tylosurus	*Tylosurus crocodilus*		+			
6	Carangiformes	Carangidae	Alepes	*Alepes kleinii*		+			
7	Carangiformes	Carangidae	Caranx	*Caranx melampygus*		+			
8	Carangiformes	Carangidae	Decapterus	*Decapterus macarellus*		+			
9	Carangiformes	Carangidae	Seriola	*Seriola dumerili*	+	+			
10	Carangiformes	Carangidae	Trachinotus	*Trachinotus blochii*	+				
11	Carangiformes	Carangidae	Trachinotus	*Trachinotus ovatus*	+	+			

12	Centrarchiformes	Terapontidae	Terapon	*Terapon theraps*		+			
13	Chaetodontiformes	Leiognathidae	Deveximentum	*Deveximentum indicium*	+				
14	Chaetodontiformes	Leiognathidae	Nuchequula	*Nuchequula nuchalis*	+				
15	Chaetodontiformes	Leiognathidae	Photopectoralis	*Photopectoralis bindus*	+				
16	Cichliformes	Cichlidae	Sarotherodon	*Sarotherodon melanotheron*	+				
17	Clupeiformes	Clupeidae	Clupanodon	*Clupanodon thrissa*	+	+			
18	Clupeiformes	Engraulidae	Coilia	*Coilia grayii*	+	+			
19	Clupeiformes	Engraulidae	Coilia	*Coilia mystus*		+			
20	Clupeiformes	Engraulidae	Coilia	*Coilia nasus*	+	+		+	
21	Clupeiformes	Engraulidae	Engraulis	*Engraulis japonicus*	+	+	+	+	
22	Clupeiformes	Clupeidae	Konosirus	*Konosirus punctatus*	+		+	+	
23	Clupeiformes	Clupeidae	Sardinella	*Sardinella hualiensis*	+	+			
24	Clupeiformes	Engraulidae	Setipinna	*Setipinna taty*	+		+	+	

25	Clupeiformes	Engraulidae	Thryssa	*Thryssa kammalensis*	+		+	+	
26	Clupeiformes	Engraulidae	Thryssa	*Thryssa vitrirostris*	+				
27	Clupeiformes	Clupeidae	Macrura	*Tenualosa reevesii*			+		
28	Ephippiformes	Ephippidae	Platax	*Platax teira*	+				
29	Gerreiformes	Gerreidae	Gerres	*Gerres oyena*		+			
30	Gobiiformes	Gobiidae	Amblychaeturichthys	*Amblychaeturichthys hexanema*	+		+	+	
31	Gobiiformes	Gobiidae	Chaeturichthys	*Chaeturichthys stigmatias*	+	+	+	+	
32	Gobiiformes	Gobiidae	Ctenotrypauchen	*Ctenotrypauchen chinensis*		+	+		
33	Gobiiformes	Gobiidae	Odontamblyopus	*Odontamblyopus lacepedii*	+		+		
34	Gobiiformes	Gobiidae	Oxyurichthys	*Oxyurichthys auchenolepis*	+	+			
35	Gobiiformes	Gobiidae	Parachaeturichthys	*Parachaeturichthys polynema*		+			
36	Gobiiformes	Gobiidae	Taenioides	*Taenioides cirratus*		+			
37	Gobiiformes	Acanthogobius	Gobionellinae	*Acanthogobius flavimanus*				+	

38	Gobiiformes	Acanthogobius	Gobionellinae	*Acanthogobius ommaturus*				+	
39	Gobiiformes	Gobiidae	Ctenotrypauchen	*Ctenotrypauchen microcephalus*				+	
40	Gobiiformes	Gobiidae	Myersina	*Myersina filifer*			+		
41	Gobiiformes	Gobiidae	Tridentiger	*Tridentiger barbatus*			+		
42	Gobiiformes	Gobiidae	Tridentiger	*Tridentiger trigonocephalus*			+		
43	Kurtiformes	Apogonidae	Jaydia	*Jaydia striatodes*		+			
44	Labriformes	Labridae	Cetoscarus	*Cetoscarus bicolor*		+			
45	Labriformes	Labridae	Chlorurus	*Chlorurus microrhinos*		+			
46	Labriformes	Labridae	Halichoeres	*Halichoeres nigrescens*		+			
47	Labriformes	Labridae	Thalassoma	*Thalassoma bifasciatum*		+			
48	Lophiiformes	Lophiidae	Lophius	*Lophius litulon*	+		+	+	
49	Lutjaniformes	Haemulidae	Plectorhinchus	*Plectorhinchus chaetodonoides*		+			
50	Mugiliformes	Mugilidae	Mugil	*Mugil cephalus*	+				

51	Mugiliformes	Mugilidae	Planiliza	*Planiliza haematocheilus*		+			
52	Mugiliformes	Mugilidae	Mugil	*Mugil soiuy*				+	
53	Osmeriformes	Osmeridae	Hypomesus	*Hypomesus nipponensis*	+	+			
54	Osmeriformes	Salangidae	Salanx	*Salanx ariakensis*	+				
55	Perciformes	Pomacentridae	Abudefduf	*Abudefduf hoefleri*	+				
56	Perciformes	Pomacentridae	Abudefduf	*Abudefduf sexfasciatus*	+				
57	Perciformes	Pomacentridae	Abudefduf	*Abudefduf sordidus*	+	+			
58	Perciformes	Sciaenidae	Collichthys	*Collichthys lucidus*	+	+	+		
59	Perciformes	Sciaenidae	Dendrophysa	*Dendrophysa russelii*	+	+			
60	Perciformes	Sciaenidae	Johnius	*Johnius grypotus*	+	+			
61	Perciformes	Sciaenidae	Johnius	*Johnius trewavasae*		+			
62	Perciformes	Sciaenidae	Larimichthys	*Larimichthys crocea*	+	+			
63	Perciformes	Sciaenidae	Larimichthys	*Larimichthys polyactis*	+		+		

64	Perciformes	Stichaeidae	Lumpenella	*Lumpenella longirostris*	+				
65	Perciformes	Menidae	Mene	*Mene maculata*		+			
66	Perciformes	Sciaenidae	Pennahia	*Pennahia pawak*		+			
67	Perciformes	Pholidae	Pholis	*Pholis fangi*	+	+	+	+	
68	Perciformes	Serranidae	Plectropomus	*Plectropomus leopardus*		+			
69	Perciformes	Pomacanthidae	Pomacanthus	*Pomacanthus xanthometopon*		+			
70	Perciformes	Siganidae	Siganus	*Siganus fuscescens*	+	+			
71	Perciformes	Siganidae	Siganus	*Siganus spinus*		+			
72	Perciformes	Siganidae	Siganus	*Siganus sutor*	+	+			
73	Perciformes	Sillaginidae	Sillago	*Sillago sihama*	+				
74	Perciformes	Ammodytidae	Ammodytes	*Ammodytes beniteguri*				+	
75	Perciformes	Sciaenidae	Argyrosomus	*Argyrosomus argentatus*			+	+	
76	Perciformes	Callionymidae	Callionymus	*Callionyuus richardsion*			+	+	

77	Perciformes	Serranidae	Lateolabrax	*Lateolabrax maculatus*				+	
78	Perciformes	Stromateidae	Pampus	*Pampus argenteus*			+	+	
79	Perciformes	Leiognathidae	Secuter	*Secuter ruconius*			+		
80	Perciformes	Zoarcidae	Zoarces	*Zoarces viviparus*				+	
81	Pleuronectiformes	Cynoglossidae	Cynoglossus	*Cynoglossus joyneri*	+		+	+	
82	Pleuronectiformes	Cynoglossidae	Cynoglossus	*Cynoglossus robustus*	+				
83	Pleuronectiformes	Pleuronectidae	Pleuronichthys	*Pleuronichthys cornutus*	+				
84	Pleuronectiformes	Pleuronectidae	Pleuronectinae	*Cleisthenes herzensteini*			+	+	
85	Pleuronectiformes	Pleuronectidae	Kareius	*Kareius bicoloratus*				+	
86	Rajiformes	Rajidae	Raja	*Raja pulchra*				+	
87	Scombriformes	Bramidae	Brama	*Brama dussumieri*		+			
88	Scombriformes	Scombridae	Scomber	*Scomber japonicus*		+		+	
89	Scombriformes	Scombridae	Scomberomorus	*Scomberomorus niphonius*		+			

90	Scorpaeniformes	Cottidae	Hemitripterus	*Hemitripterus villosus*				+	
91	Scorpaeniformes	Hexagrammidae	Hexagrammos	*Hexagrammos otakii*	+		+	+	
92	Scorpaeniformes	Liparidae	Liparis	*Liparidae Liparis*			+		
93	Scorpaeniformes	Liparidae	Liparis	*Liparis tanakae*	+	+	+	+	
94	Scorpaeniformes	Platycephalidae	Platycephalus	*Platycephalus indicus*			+	+	
95	Scorpaeniformes	Sebastidae	Sebastes	*Sebastes schlegeli*				+	
96	Spariformes	Sparidae	Acanthopagrus	*Acanthopagrus latus*		+			
97	Spariformes	Nemipteridae	Nemipterus	*Nemipterus japonicus*	+				
98	Spariformes	Sparidae	Rhabdosargus	*Rhabdosargus sarba*		+			
99	Spariformes	Nemipteridae	Scolopsis	*Scolopsis vosmeri*	+				
100	Syngnathiformes	Callionymidae	Callionymus	*Callionymus valenciennei*	+	+			
101	Syngnathiformes	Syngnathidae	Phycodurus	*Phycodurus eques*	+	+			
102	Syngnathiformes	Callionymidae	Repomucenus	*Repomucenus ornatipinnis*	+				

103	Syngnathiformes	Syngnathidae	Syngnathus	*Syngnathus schlegeli*	+				
104	Syngnathiformes	Syngnathidae	Syngnathus	*Syngnathus typhle*	+				
105	Syngnathiformes	Syngnathidae	Syngnathus	*Syngnathus acus*			+		
106	Tetraodontiformes	Balistidae	Balistapus	*Balistapus undulatus*		+			
107	Tetraodontiformes	Tetraodontidae	Lagocephalus	*Lagocephalus spadiceus*		+			
108	Tetraodontiformes	Tetraodontidae	Takifugu	*Takifugu bimaculatus*	+				
109	Uranoscopiformes	Ammodytidae	Ammodytes	*Ammodytes hexapterus*		+			
110	Uranoscopiformes	Ammodytidae	Ammodytes	*Ammodytes japonicus*		+			
Total	28	51	89	110	51	56	28	29	
