# Supplementary material for: Unraveling Fish Community Diversity and Structure in the Yellow Sea: Evidence from Environmental DNA Metabarcoding and Bottom Trawling
Source: Animals (Basel). 2025 Apr 30;15(9):1283. doi: 10.3390/ani15091283 (PMC12070852; doi:10.3390/ani15091283)
Supplement: Supplementary file 1 [file animals-15-01283-s001.zip › Supplementary Table S3.pdf]

Supplementary Table S3: Species traits used to calculate functional diversity.

Species	Max Length (cm)	Depth Range (m)	Trophic Level	Repro. Mode	Repro. Fertil	Repro. Parent Care	Environment	Body shape
Amblychaeturichthys hexanema	17.4	30	3.4	dioecism	external	none	demersal	elongated
Chaeturichthys stigmatias	28.2	30	3.8	dioecism	external	none	demersal	elongated
Coilia nasus	41	50	3.0	dioecism	external	none	pelagic-neritic	elongated
Collichthys lucidus	19.8	87	3.6	dioecism	external	none	demersal	fusiform
Ctenotrypauchen chinensis	19.2	20	3.8	dioecism	external	none	demersal	elongated
Cynoglossus joyneri	39.4	50	4.3	dioecism	external	none	demersal	flat
Engraulis japonicus	18.0	400	3.1	dioecism	external	none	pelagic-neritic	elongated
Hexagrammos otakii	57.0	16	3.8	dioecism	external	none	demersal	fusiform
Konosirus punctatus	32.0	10	2.9	dioecism	external	none	pelagic-neritic	fusiform
Larimichthys polyactis	40.0	108	3.7	dioecism	external	none	benthopelagic	fusiform
Liparis tanakae	56.3	71	4.3	dioecism	external	none	demersal	elongated
Lophius litulon	150	535	4.1	dioecism	external	none	bathydemersal	short
Odontamblyopus lacepedii	30.3	6	3.9	dioecism	external	none	benthopelagic	elongated
Pholis fangi	16.8	30	3.2	dioecism	external	none	demersal	elongated

Scomber japonicus	64.0	300	3.4	dioecism	external	none	pelagic-neritic	fusiform
Setipinna taty	15.3	50	3.6	dioecism	external	none	pelagic-neritic	elongated
Thryssa kammalensis	18.0	20	3.4	dioecism	external	none	pelagic-neritic	elongated
Abudefduf hoefleri	20.0	15	2.7	dioecism	external	none	reef-associated	short
Abudefduf sexfasciatus	19.0	19	2.7	dioecism	external	none	reef-associated	short
Abudefduf sordidus	24.0	3	2.9	dioecism	external	none	reef-associated	short
Acanthopagrus latus	40.0	50	3.8	dioecism	external	none	demersal	fusiform
Alepes kleinii	18.2	95	3.5	dioecism	external	none	reef-associated	fusiform
Ammodytes hexapterus	30.0	275	3.1	dioecism	external	none	benthopelagic	elongated
Balistapus undulatus	30	60	3.4	dioecism	external	none	reef-associated	fusiform
Brama dussumieri	22.5	299	3.8	dioecism	external	none	pelagic-neritic	flat
Callionymus valenciennei	17.4	30	3.3	dioecism	external	none	demersal	elongated
Caranx melampygus	117.0	190	4.5	dioecism	external	none	reef-associated	fusiform
Cetoscarus bicolor	50	29	2.0	dioecism	external	none	reef-associated	fusiform
Clupanodon thrissa	26.0	50	3.1	dioecism	external	none	pelagic-neritic	fusiform
Coilia grayii	33.0	50	3.4	dioecism	external	none	pelagic-neritic	elongated
Coilia mystus	21.0	20	3.2	dioecism	external	none	pelagic-neritic	elongated

Ctenochaetus tominiensis	16.0	45		dioecism	external	none	reef-associated	flat
Cynoglossus robustus	40.0	95	3.5	dioecism	external	none	demersal	flat
Decapterus macarellus	46.0	400	4.0	dioecism	external	none	pelagic-oceanic	fusiform
Dendrophysa russelii	38.3	235	3.6	dioecism	external	none	demersal	fusiform
Deveximentum indicium	8.8	15	3.0	dioecism	external	none	pelagic-neritic	flat
Gerres oyena	30.0	20	2.7	dioecism	external	none	reef-associated	fusiform
Gymnothorax javanicus	300.0	50	3.9	dioecism	external	none	reef-associated	elongated
Halichoeres nigrescens	14.6	7	3.4	dioecism	external	none	reef-associated	fusiform
Hypomesus nipponensis	17.0	30	3.3	dioecism	external	none	pelagic	elongated
Jaydia striatodes	10.0	35	3.5	dioecism	external	none	reef-associated	fusiform
Johnius grypotus	13.7	85	3.4	dioecism	external	none	benthopelagic	fusiform
Johnius trewavasae	22.0	39	3.5	dioecism	external	none	benthopelagic	fusiform
Lagocephalus spadiceus	37.4	82	3.7	dioecism	external	none	demersal	fusiform
Larimichthys crocea	80.0	120	3.7	dioecism	external	none	benthopelagic	fusiform
Lumpenella longirostris	42.0	1115	3.1	dioecism	external	none	demersal	elongated
Mene maculata	30.0	150	3.5	dioecism	external	none	reef-associated	flat
Mugil cephalus	100.0	120	2.5	dioecism	external	none	benthopelagic	fusiform

Nemipterus japonicus	34.0	75	4.1	dioecism	external	none	demersal	fusiform
Nuchequula nuchalis	25.0	8	3.0	dioecism	external	none	pelagic-neritic	fusiform
Oxyurichthys auchenolepis	13.0	29	3.7	dioecism	external	none	demersal	elongated
Parachaeturichthys polynema	15.0	30	3.1	dioecism	external	none	demersal	elongated
Pennahia pawak	23.2	47	3.3	dioecism	external	none	benthopelagic	fusiform
Photopectoralis bindus	14.0	158	2.9	dioecism	external	none	demersal	flat
Phycodurus eques	35.0	26	3.3	dioecism	external	none	reef-associated	elongated
Pisodonophis cancrivorus	108.0	19	3.8	dioecism	external	none	reef-associated	elongated
Planiliza haematocheilus	80.0	5	2.5	dioecism	external	none	pelagic-neritic	fusiform
Platax teira	70.0	22	4.0	dioecism	external	none	reef-associated	flat
Plectorhinchus chaetodonoides	72.0	29	3.8	dioecism	external	none	reef-associated	fusiform
Plectropomus leopardus	120.0	97	4.4	dioecism	external	none	reef-associated	fusiform
Pleuronichthys cornutus	30.0	168	3.2	dioecism	external	none	demersal	flat
Pomacanthus xanthometopon	38.0	25	2.7	dioecism	external	none	reef-associated	flat
Repomucenus ornatipinnis	17.0	5	3.4	dioecism	external	none	demersal	elongated
Rhabdosargus sarba	80.0	60	3.3	dioecism	external	none	reef-associated	fusiform

Salanx ariakensis	14.7	8	2.8	dioecism	external	none	demersal	elongated
Sardinella hualiensis	12.5	50	2.9	dioecism	external	none	pelagic-neritic	fusiform
Sarotherodon melanotheron	28.0	3	2.5	dioecism	external	none	demersal	fusiform
Scolopsis vosmeri	25.0	23	3.5	dioecism	external	none	reef-associated	fusiform
Scomberomorus niphonius	113.0	200	4.5	dioecism	external	none	pelagic-neritic	fusiform
Seriola dumerili	190.0	384	4.5	dioecism	external	none	reef-associated	fusiform
Siganus fuscescens	40.0	49	2.0	dioecism	external	none	reef-associated	fusiform
Siganus spinus	28.0	49	2.0	dioecism	external	none	reef-associated	fusiform
Siganus sutor	45.0	49	2.3	dioecism	external	none	reef-associated	fusiform
Sillago sihama	31.0	60	3.3	dioecism	external	none	reef-associated	elongated
Syngnathus schlegeli	30.0	15	3.1	dioecism	external	none	demersal	elongated
Syngnathus typhle	35.0	19	4.3	dioecism	external	none	demersal	elongated
Taenioides cirratus	30.0	6	3.9	dioecism	external	none	demersal	elongated
Takifugu bimaculatus	30.0		3.4	dioecism	external	none	demersal	fusiform
Terapon theraps	30.0	10	3.5	dioecism	external	none	demersal	fusiform
Thalassoma bifasciatum	25.0	40	3.3	dioecism	external	none	reef-associated	short
Thryssa kammalensis	18.0	20	3.4	dioecism	external	none	pelagic-neritic	elongated

Thryssa vitrirostris	22.0	50	3.4	dioecism	external	none	demersal	fusiform
Trachinotus blochii	110.0	7	3.7	dioecism	external	none	demersal	flat
Trachinotus ovatus	70.0	200	3.7	dioecism	external	none	demersal	fusiform
Tylosurus crocodilus	150.0	13	4.4	dioecism	external	none	demersal	elongated
Acanthogobius_flavimanus	30	5	3.4	dioecism	external	none	demersal	elongated
Acanthogobius_ommaturus	43	10	3.4	dioecism	external	none	demersal	elongated
Ammodytes_beniteguri	22.7	170	3.3	dioecism	external	none	demersal	elongated
Argyrosomus_argentatus	40	140	4.1	dioecism	external	none	benthopelagic	fusiform
Callionymus_richardsoni	20.4	100	3.2	dioecism	external	none	demersal	short
Cleisthenes_herzensteini	47	200	3.4	dioecism	external	none	demersal	flat
Conger_japonicus	140	140	4	dioecism	external	none	demersal	elongated
Ctenotrypauchen_microcephalus	18	10	3.7	dioecism	external	none	demersal	short
Hemitripterus_villosus	35	550	4.3	dioecism	external	none	demersal	elongated
Kareius_bicoloratus	50	150	3.7	dioecism	external	none	demersal	flat
Lateolabrax_maculatus	102	149	3.1	dioecism	external	none	demersal	elongated
Liparidae_Liparis	15	300	3.6	dioecism	external	none	demersal	elongated

Mugil_soiuy	80	10	2.5	dioecism	external	none	demersal	elongated
Myersina_filifer	13.2	25	3.4	dioecism	external	none	demersal	elongated
Pampus_argenteus	60	110	3.3	dioecism	external	none	benthopelagic	flat
Platycephalus_indicus	100	200	3.6	dioecism	external	none	demersal	elongated
Raja_pulchra	115	120	4.4	dioecism	external	none	demersal	flat
Sebastes_schlegeli	65	100	4.1	dioecism	external	none	demersal	elongated
Secuter_ruconius	8	60	2.7	dioecism	external	none	demersal	fusiform
Syngnathus_acus	50	110	3.3	dioecism	external	none	demersal	elongated
Tenualosa_reevesii	61.6	50	3.2	dioecism	external	none	demersal	fusiform
Tridentiger_barbatus	10.4	29	3.5	dioecism	external	none	demersal	elongated
Tridentiger_trigonocephalus	11	30	3.4	dioecism	external	none	demersal	elongated
Zoarces_viviparus	52	40	3.5	dioecism	external	none	demersal	elongated
