# Supplementary material for: Unraveling Fish Community Diversity and Structure in the Yellow Sea: Evidence from Environmental DNA Metabarcoding and Bottom Trawling
Source: Animals (Basel). 2025 Apr 30;15(9):1283. doi: 10.3390/ani15091283 (PMC12070852; doi:10.3390/ani15091283)
Supplement: Supplementary file 1 [file animals-15-01283-s001.zip › Supplementary Table S4.pdf]

Supplementary Table S4: Summary of Alpha diversity indices

Station	chao1		Shannon		Simpson		Pielou_J		Station	chao1		Shannon		Simpson		Pielou_J	
LYG	E-LYG	D-LYG	E-LYG	D-LYG	E-LYG	D-LYG	E-LYG	D-LYG	ZH	E-ZH	D-ZH	E-ZH	D-ZH	E-ZH	D-ZH	E-ZH	D-ZH
1	85	19	3.001	1.902	0.905	0.749	0.676	0.671	1	33	5	2.451	1.386	0.831	0.715	0.701	0.861
2	60	16	2.019	1.959	0.815	0.807	0.493	0.723	2	31	7	2.099	1.696	0.859	0.800	0.611	0.946
3	49	21.333	2.343	2.122	0.757	0.817	0.602	0.734	3	36	6	0.308	1.550	0.092	0.776	0.086	0.963
4	26	10	1.643	1.832	0.764	0.801	0.504	0.796	4	24	6	2.361	0.588	0.894	0.250	0.743	0.328
5	45	19	1.541	1.821	0.630	0.801	0.405	0.710	5	59	30	3.106	0.524	0.945	0.195	0.762	0.238
6	46	17	1.930	2.411	0.746	0.879	0.504	0.851	6	30	8	2.086	1.065	0.853	0.506	0.613	0.547
7	40	11.333	1.178	0.822	0.456	0.331	0.319	0.343	7	22	6.5	1.869	1.159	0.773	0.598	0.605	0.647
8	22	11.333	1.247	1.219	0.480	0.506	0.403	0.508	8	47	14	3.093	1.631	0.944	0.768	0.803	0.680
9	35	11	2.033	1.435	0.771	0.587	0.572	0.598	9	53	8	3.043	1.383	0.946	0.712	0.766	0.711
10	40	14.333	2.173	2.026	0.807	0.802	0.589	0.768	12	60	6	1.652	1.172	0.751	0.622	0.404	0.654
11	27	15.25	1.251	1.973	0.498	0.778	0.380	0.729	13	54	5	3.261	1.182	0.956	0.603	0.817	0.734
12	23	15.5	1.565	1.775	0.601	0.751	0.499	0.673	15	40	6	3.382	1.306	0.962	0.669	0.917	0.729
13	23	10	1.836	1.208	0.763	0.545	0.585	0.550	16	57	9	2.765	1.152	0.921	0.541	0.684	0.643
14	25	29	1.869	1.459	0.759	0.674	0.581	0.553	17	70	4	3.199	1.311	0.935	0.716	0.753	0.946
15	30	11	2.044	2.053	0.804	0.846	0.601	0.856	18	53	16	2.841	1.490	0.926	0.701	0.716	0.621
16	31	16	1.904	1.821	0.768	0.756	0.554	0.673	19	42	7	2.252	1.550	0.846	0.757	0.602	0.797
