# Supplementary material for: Unraveling Fish Community Diversity and Structure in the Yellow Sea: Evidence from Environmental DNA Metabarcoding and Bottom Trawling
Source: Animals (Basel). 2025 Apr 30;15(9):1283. doi: 10.3390/ani15091283 (PMC12070852; doi:10.3390/ani15091283)
Supplement: Supplementary file 1 [file animals-15-01283-s001.zip › Supplementary Table S5.pdf]

Supplementary Table S5: Monte Carlo permutation test for RDA (Conditional effect) based on eDNA metabarcoding data (A) and bottom trawling data (B).

A

Explanatory variable	R^2	Adujsted R^2	Contribution (%)	F	*P*
Temp	33.5961	27.6451	54.1	15.2	0.002**
Nitrate	10.6812	8.7892	17.2	5.6	0.002**
Ammonium	9.2529	7.6139	14.9	5.6	0.008**
Nitrite	4.1607	3.4237	6.7	2.7	0.08
Sal	2.4219	1.9929	3.9	1.6	0.172
DO	1.3041	1.0731	2.1	0.8	0.41
Phosphate	0.621	0.511	1	0.4	0.72
First Axis				13.7	0.002
All Axes	62.04	51.05		5.6	0.002

B

Explanatory variable	R^2	Adujsted R^2	Contribution (%)	F	*P*
Temp	10.6829	3.9766	33.7	3.6	0.002**
Nitrate	7.1008	2.6432	22.4	2.5	0.024*
Ammonium	6.6253	2.4662	20.9	2.5	0.02*
Sal	2.4726	0.9204	7.8	0.9	0.528
Nitrite	2.0922	0.7788	6.6	0.8	0.596
DO	1.8703	0.6962	5.9	0.7	0.632
Phosphate	0.8559	0.3186	2.7	0.3	0.9
First Axis				11.4	0.092
All Axes	31.7	11.8		2.4	0.084

Note：“NS”: not significant, **: $p < 0.01$, *: $p < 0.05$.
