# Supplementary material for: Unraveling Fish Community Diversity and Structure in the Yellow Sea: Evidence from Environmental DNA Metabarcoding and Bottom Trawling
Source: Animals (Basel). 2025 Apr 30;15(9):1283. doi: 10.3390/ani15091283 (PMC12070852; doi:10.3390/ani15091283)
Supplement: Supplementary file 1 [file animals-15-01283-s001.zip › Supplementary Table S6.pdf]

Supplementary Table S6: Summary of the biodiversity indicators, taxonomic richness, phylogenetic diversity, and functional diversity, measured by eDNA and bottom trawling at each sampling station.

Station	**Taxonomic richness**		**Phylogenetic diversity**		**Functional diversity**	
	eDNA	Bottom trawling	eDNA	Bottom trawling	eDNA	Bottom trawling
LYG1	41	17	6.63--	6.10	7.21	6.00
LYG2	30	15	5.96	6.41	6.00	5.91
LYG3	33	18	5.95	6.36++	6.27	5.43
LYG4	31	10	5.84	5.61++	5.89	3.89
LYG5	33	13	5.81	4.08	6.12	4.88
LYG6	28	17	5.56	4.64	5.82	5.65
LYG7	31	11	5.73	3.95	5.92	4.63
LYG8	26	11	5.1-	3.64	5.41	4.72
LYG9	29	11	5.07	3.81	6.52	4.60
LYG10	28	14	5.55++	4.35	5.23	6.00
LYG11	22	15	4.63-	4.53	4.72	7.35++
LYG12	26	14	5.35	5.98++	5.03	5.32
LYG13	25	9	5.52	3.30	5.01	3.71
LYG14	24	14	5.43	5.35	4.95	3.79
LYG15	27	11	5.31	5.17	4.96	3.00--
LYG16	28	15	5.69	4.21	5.18	5.37
ZH1	17	5	5.13++	1.54	5.64	3.37
ZH2	16	6	4.31	1.56	5.41	3.22
ZH3	23	5	5.58+	1.22	5.87	2.84-
ZH4	18	6	4.06	1.86	5.09	3.40
ZH5	27	9	4.78	2.34	5.49	4.80
ZH6	18	7	4.87	1.89	4.83	4.37
ZH7	21	6	4.13++	1.82	4.91	3.84
ZH8	26	11	5.01	2.52	5.32	4.88
ZH9	24	10	4.37-	2.22	5.32	4.31
ZH12	30	8	4.93--	2.01	6.21	3.56
ZH13	27	6	5.00	1.12	5.76	2.10--
ZH15	25	11	5.45	2.33	6.23	4.84
ZH16	30	7	5.12--	1.91	5.61	3.29
ZH17	30	8	5.63	1.58	5.57	3.96
ZH18	25	6	4.82	1.56	5.75	4.32+
ZH19	31	14	6.85+	3.52	6.05	5.63

Note: For phylogenetic and functional diversity, significant standardized effect sizes (SES) showing over-dispersion (+) or clustering (−) are indicated. (+), over dispersion 90%; (++), over dispersion 95%; (-), over clustering 90%; (--), over clustering 95%.
