# Supplementary figures and images for: Unraveling Fish Community Diversity and Structure in the Yellow Sea: Evidence from Environmental DNA Metabarcoding and Bottom Trawling
Source: Animals (Basel). 2025 Apr 30;15(9):1283. doi: 10.3390/ani15091283 (PMC12070852; doi:10.3390/ani15091283)

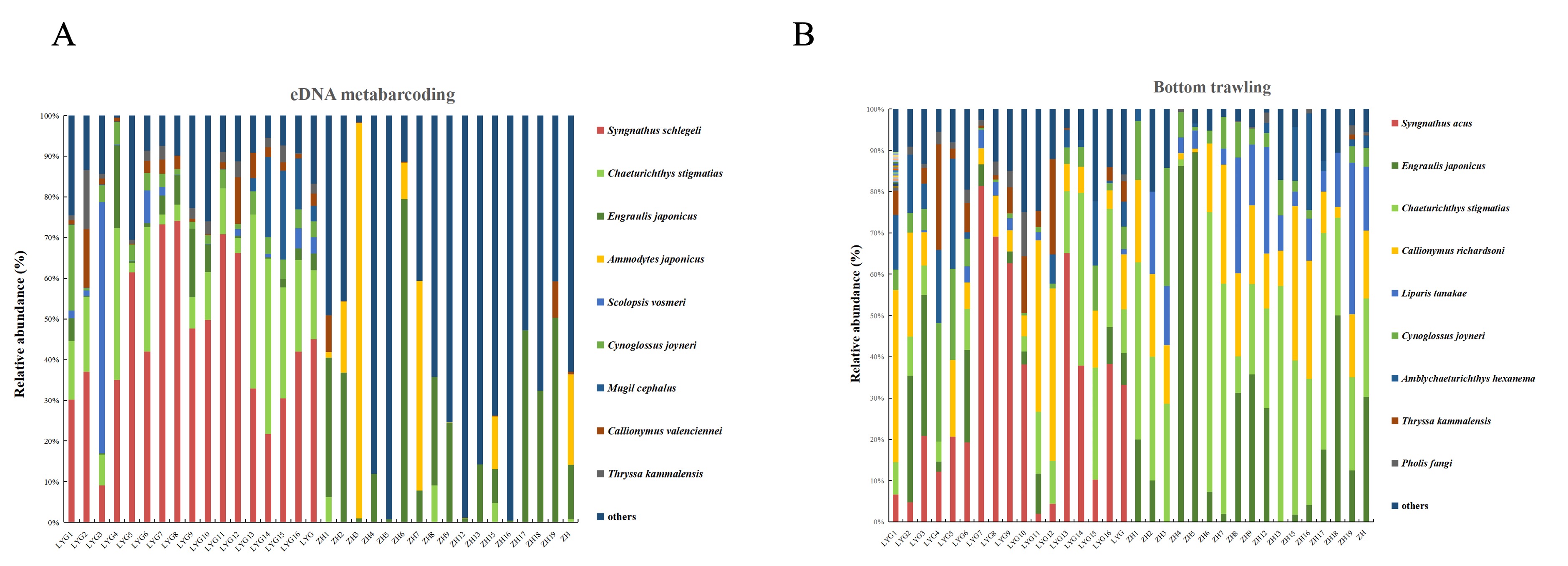

Supplement: Supplementary file 1 [file animals-15-01283-s001.zip › Figure S1.jpg]
